# 

**Published:** 1891-09

**Authors:** 


					rp    SEPTEMBER, 1891.
Vol. IX. No. 33.
THE
BRISTOL
medico-chirurgical
JOURNAL
& <tarterlB Journal of tbe dibeMcal Sciences for tbe "WHest
of England anfc Soutb Males
PUBLISHED UNDER THE AUSPICES OF
the BRISTOL MEDICO-CHIRURGICAL SOCIETT.
Tiitib ? . y- ?XvV''4'
/ v" O <Q -41 /S r\ r\
EDITOR: ,.t) "j (/,
J. GREIG SMITH, M.A., F.R.S.E.
ASSOCIATED WITH . [
Barclay j. baron, m.b., c.m. f. r. cross, m.b.
J- MICHELL CLARKE, M.B., M.A. W. H. HARSANT.
ASSISTANT-EDITOR :
L. M. GRIFFITHS.
"Scire est riesct're, nisi i& me
Scire alius sciret."
BRISTOL: J. W. ARROWSMITH;
London; J. & A. Churchill, 11 New Burlington Street.
Price One Shilling and Sixpence.

				

